# Reol: R interface to the Encyclopedia of Life

**DOI:** 10.1002/ece3.1109

**Published:** 2014-05-26

**Authors:** Barbara L Banbury, Brian C O'Meara

**Affiliations:** Department of Ecology and Evolutionary Biology, University of TennesseeKnoxville, Tennessee

**Keywords:** Application programming interface, Encyclopedia of Life, programmatic access, reproducible research, taxonomy

## Abstract

The Encyclopedia of Life is a website that hosts information about life on Earth. Its mission is to increase awareness and understanding of living nature through a freely accessible digital source. Information is publicly available through graphical webpages (browser interface) or through an application programming interface (API). We developed *Reol*, an open-source package for the R environment, which downloads data from the EOL API, searches for and extracts specific information, and builds tables with quantitative data and/or hierarchical classifications. We provide a detailed description how *Reol* can be used as a bridge between the R environment and the EOL API to extract quantitative or hierarchical content. It will be particularly useful for researchers who want information about taxonomic groups of interest (for example: how much information is known about flatworm species? What are the taxonomic synonyms for bird species?) or construct a taxonomic tree. *Reol* is a tool for researchers who wish to download and gather data from EOL or its provider pages. We provide numerous functions within R for downloading, gathering data in different forms, creating taxonomic trees, and plotting data, which work with functions already available through various packages. It joins a growing body of R packages that interact with web-based APIs to streamline data acquisition, thereby easing the analysis of large publicly available datasets.

## Introduction

The Encyclopedia of Life (http://eol.org/) is a website dedicated to preserving information about life on Earth. It was developed in 2007 to host “a webpage for every species”; however, it has expanded to include information from resources such as museums, taxon-specific societies, and scientists, about all taxonomic groups (species and higher rankings). It brings together a community of professional scientists, educators, students, and the interested public.

Page content comes in forms such as text, maps, multimedia, common names, and scientific synonyms. It is built from multiple sources, including over 250 organizations that have agreed to contribute content (http://eol.org/content_partners) and twelve providers that contribute hierarchical classification data (http://eol.org/info/classification_providers). Data are curated by a community of EOL members and experts who organize, rate, and authenticate new content. Any EOL member can become a curator, but those members with taxonomic expertise are given extended privileges (for example, changing the review status of EOL materials).

The main interface is the user-friendly website. It is easy for users to point and click through each taxon's table of contents and view information or download various media. However, behind the scenes of the graphical user interface (GUI) is the application programming interface (API), which is standard allowing software to communicate with other software. The API provides a mechanism for viewing text representations of EOL page content.

The R package *Reol* provides a link between the EOL API and the R environment. Text from these pages is downloaded, organized for content, and amassed into a tabular format. R (R Development Core Team 2012) is a free, open-source, cross-platform statistical language that is frequently used in biology (Paradis et al. 2004; Rabosky 2006; Harmon et al. 2008; Paradis 2011; O'Meara 2012). R packages that interact with scientific databases via APIs are also becoming increasingly common (ex. Boettiger and Lang 2012; Chamberlain et al. 2013; Winter 2013, and see http://ropensci.org for many more). There are already numerous functions available for manipulating trees and data, performing statistical analyses, and visualizing results that easily work with the functions of *Reol*.

The *Reol* package provides several key features:

1. Data Accessibility. Users gain programmatic access to download information from the Encyclopedia of Life API directly from the R environment. EOL pages are drawn into R via XML formatting and can be saved either as XML pages or as an R object.
2. Data Compilation. *Reol* extracts data from the XML pages and builds end-user data tables and trees. Some functions use EOL or provider pages only and others use both.
3. Integration and Reproducibility. *Reol* functions integrate into existing R functionality with ease. This makes data collection and tree plotting relatively easy for those with a working knowledge of R. Users can write scriptable workflows that can download and tabulate data off multiple pages and which can be rerun at any time to retrieve current results.

Users who intend to download large amounts of data are asked to identify their usage by generating a unique API key. This can be generated from a user preference page after creating and logging into an EOL account. It can be stored as an R object and added to *Reol* calls, which will automatically add them to the API call. A stable version of *Reol* can be downloaded from the Comprehensive R Archive Network (CRAN; http://cran.r-project.org/web/packages/Reol/index.html). In addition to feature requests and bug tracking, the newest version of *Reol* can be downloaded from R-Forge (https://r-forge.r-project.org/projects/reol/). We welcome new developers to the project at R-Forge to improve or extend the existing code.

## Data Accessibility

*Reol* accesses EOL through its RESTful (REpresentational State Transfer; Fielding 2000) API, which allows for search conditions within a URL (for example: http://eol.org/api/search/1.0.xml?q=Ursus). *Reol* builds the URL based on function arguments and sends it out to the API through the RCurl package (Temple Lang 2012a). There are three dedicated functions for retrieving information: one that downloads directly from an EOL unique taxon identifier (for example “14349” refers to the genus *Ursus*), one that searches the database for a taxonomic name match (either exact or fuzzy name matching), and one that searches for hierarchical provider pages. *Reol* functions read in and parse the retrieved XML (extensible markup language) formatted pages into meaningful R objects using the XML package in R (Temple Lang 2012b). The retrieved data can be saved in two ways; either individual pages can be saved as separate XML files or they can be saved together as an R object with each entry in the list being associated with a single query. For instance, the following code will retrieve the EOL page on *Ursus*:

Ursus1 < – DownloadEOLpages(14349, to.file = FALSE)

Ursus2 < – DownloadSearchedTaxa(“Ursus”, to.file = FALSE, exact = TRUE)

PB < – DownloadSearchedTaxa(“Ursus maritimus”, to.file = FALSE, exact = TRUE)

Even though different in approach, the first two functions will download the exact same page. In both cases, the XML data will be imported into R as an object and can be saved to the workspace or using base R's save function. The alternative is to change the to.file argument to TRUE, and it will save the XML file in the working directory.

Users can also download provider pages to gain access to hierarchical taxonomic information via an EOL provider. To download these pages, a user will have to choose one of the available providers. There are several *Reol* functions to help determine which providers contribute page content (ProviderCount and BestProvider); however, these functions do not take into account what type of content each provider is contributing. This means that one provider may only provide a taxonomic synonyms list and no hierarchical information, so if a user does not find the desired information, it may be worthwhile to choose a different provider.

NCBI.Ursus < – DownloadHierarchy(Ursus1, database = “NCBI Taxonomy”, to.file = FALSE)

GBIF.Ursus < – DownloadHierarchy(Ursus1, database = “GBIF Nub Taxonomy”, to.file = FALSE)

## Data Compilation

*Reol* has many functions to extract data from the EOL and provider pages. In most cases, *Reol* will construct end-user data that includes the taxon name, EOL or provider identification number, and whatever data are requested in tabular format. Some functions only use information of the EOL or provider pages, and some require information from both to function properly. A few key functions are outlined below and in Table 1.

**Table 1:** Key *Reol* functions

Function	Pages	Description
GetRichnessScores	EOL	An EOL composite score of data coverage
DataObjectOverview	EOL	Types and number of EOL multimedia
GetCommonNames	EOL	Taxonomic vernacular names
GetReferences	EOL	Taxonomic references used by EOL
GetIUCNStat	EOL	IUCN threat status
ProviderCount	EOL	Determines provider contribution
BestProvider	EOL	Provider with the most coverage
GatherSynonyms	Hier	Taxonomic synonyms list
TaxonParents	Hier	Taxonomic parentage
TaxonChildren	Hier	Taxonomic offspring
MakeHierarchyTree	Hier	Build a taxonomic dendrogram
MakeEdgeLabels	Hier	Create taxonomic edge labels for tree
MatchTaxatoEOLID	N/A	Searches server for unique EOL ID
MatchHierPageToEOLdata	EOL+Hier	Matches EOL and provider IDs
MatchDataToTreeTips	EOL+Hier	Matches data to the taxonomic tips

Richness Scores (GetRichnessScores): The EOL richness score is an overall metric for how much information is available for each taxon. This score is a composite of a few factors, including amount of descriptive text, number of media sources, number of provider sources that contribute information, and whether the information has been reviewed. Scores range from 0 to 100, and low scores indicate a paucity of information.

GetRichnessScores(Ursus1)

Data Objects (DataObjectOverview, GatherDataObjectInformation): EOL hosts many different kinds of media, from text descriptions to images and sound recordings. These media are each considered data objects and are individually accessioned into EOL. *Reol* has several functions that can deal with collecting information from these objects, including an overview of types and counts of media (DataObjectOverview) or gathering specific information from a single file (GatherDataObjectInformation).

DataObjectOverview(Ursus1)

Common Names (GetCommonNames): EOL hosts common or vernacular names for species. *Reol* deals with this data in two ways, either by counting the number of common names per language or by returning the name itself with the associated language.

GetCommonNames(Ursus1, output = “counts”)

GetCommonNames(Ursus1, output = “detail”)

References (GetReferences): This function returns which references EOL uses to supply taxonomic information. This information can either be in count data (raw number of references) or a detailed reference list.

GetReferences(Ursus1, output = “counts”)

GetReferences(Ursus1, output = “detail”)

IUCN Status (GetIUCNStat): This function returns the International Union for Conservation of Nature (IUCN) conservation status if provided. Data will be either not available (NA), not evaluated (NE), data deficient (DD), least concern (LC), near threatened (NT), vulnerable (VU), endangered (EN), critically endangered (CR), extinct in the wild (EW), or extinct (EX).

GetIUCNStat(PB)

Providers (ProviderCount, BestProvider): *Reol* has several functions available to help choose a provider. Providers can vary in their taxonomic coverage or content provided to EOL pages. Some providers give information regarding all of life (for example, NCBI or GBIF), while others are taxonomically focused and provide deep content on a group of organisms rather than widespread coverage (for example, Index Fungorum or The Reptile Database). A user will need to choose a provider to use when downloading hierarchy pages (see description above).

ProviderCount(Ursus1)

BestProvider(Ursus1)

Taxonomic Synonyms (GatherSynonyms): Providers can contribute a list of taxonomic synonyms, although coverage can vary. For example, NCBI does not report any synonyms for *Ursus,* but GBIF does.

GatherSynonyms(NCBI.Ursus, output = “detail”)

GatherSynonyms(GBIF.Ursus, output = “detail”)

Taxonomic Hierarchy (TaxonChildren, TaxonParents): Some providers will contribute taxonomic hierarchy information. *Reol* can find the taxonomic parentage or offspring from any taxon with this information.

TaxonParents(NCBI.Ursus)

TaxonChildren(NCBI.Ursus)

**Figure 1:**
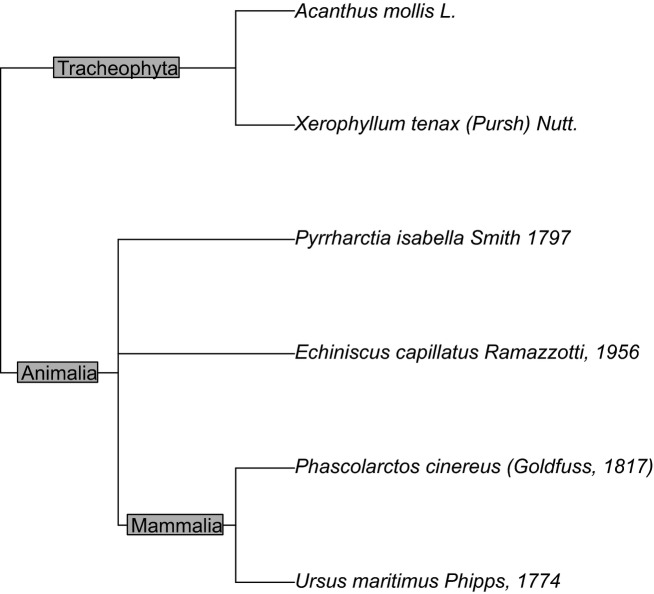
Taxonomic relationship of “bears”.

Hierarchy Trees (MakeHierarchyTree, MakeEdgeLabels): For those taxa with overlapping hierarchy information, *Reol* can create a taxonomic dendrogram (tree) and edge labeling based on classification data. Note that trees can only be constructed where data are complete. When hierarchy information comes in with gaps, the user must specify where to drop data. For example, in a set of terminal taxa of genera and species, the user will have to decide whether to drop the taxa that lack species information from the final tree or drop the rank of species and create a generic-level tree. The MakeTreeData function will show where missing data occur. (See the next section for more examples of tree building and labeling). The example below shows how relationships of disparate groups (Polar Bears, Koala Bears, Water Bears, Bear Grass, Bear's Breeches, and Wooly Bears) can be shown together (Fig. 1).

bearlist < – c(“Ursus maritimus”, “ Phascolarctos cinereus”,

“ Echiniscus capillatus”, “Xerophyllum tenax”,

“Acanthus mollis”, “Pyrrharctia Isabella”)

bears < – DownloadHierarchy(DownloadSearchedTaxa(bearlist))

MakeTreeData(bears)

bearTree < – MakeHierarchyTree(bears)

bearEdges < – MakeEdgeLabels(bears, duplicateEdgeLabels = “recent”)

plot(bearTree)

edgelabels(text = names(bearEdges), edge = bearEdges, bg = “DarkGray”)

Matching functions (MatchTaxatoEOLID, MatchHierPageToEOLdata, MatchDataToTreeTips): Several *Reol* functions ease transition between types of EOL data. These functions search through EOL or provider identifications and match taxon names, as taxon names are not standardized among providers. MatchTaxatoEOLID will search EOL for the EOL identification number associated with a search term. MatchHierPageToEOLdata matches EOL and provider identifications. Finally, MatchDataToTreeTips matches any data (EOL or provider) to tip taxa on a hierarchy tree to ease plotting data on a tree. More tree examples are below.

MatchTaxatoEOLID(“Ursus”, exact = TRUE)

MatchHierPageToEOLdata(NCBI.Ursus, GetRichnessScores(Ursus1)

MatchDataToTreeTips(bearTree, GatherSynonyms(bears, “c”)

## Integration and Reproducibility

*Reol* functions can operate independently or integrate into workflows of those already working within the R environment. As EOL data are constantly being added to the database, batch scripts can be written and rerun at any time to retrieve the most up-to-date data. This includes new taxonomic revisions from providers or newly accessioned EOL content. We provide the following examples of some of the functionality that *Reol* offers; all code from the examples can be found in the Appendix S1.

**Figure 2:**
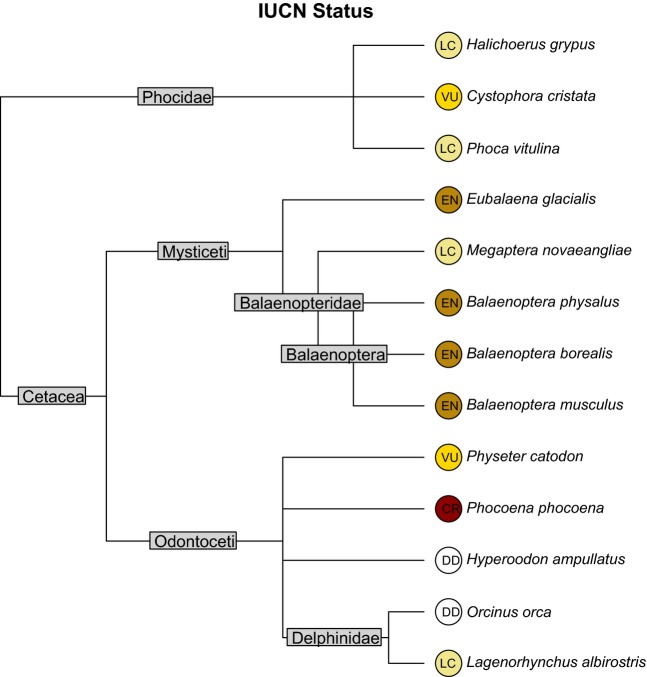
From Example 1, a tree showing the taxonomic relationships of Icelandic marine mammals.

Example 1: A researcher studies marine mammals off the coast of Iceland. She was already working with the Global Biodiversity Information Facility (GBIF, http://www.gbif.org/) to map occurrences within the R environment (*rgbif*, Chamberlain et al. 2013) and would like to combine some of this information with information from the EOL Web site. She first uses *Reol* to download the EOL pages for the thirteen species of mammals that visit the Icelandic coasts. Using the EOL pages, she gathers their IUCN statuses and then downloads the matching provider pages from the NCBI catalog. She uses the provider pages to build a taxonomic tree and plots IUCN statuses (see Fig. 2).

Example 2: A teacher in France wants his students to find information about introduced species. He plans to give each student a species with a little starting information to get them going, such as common names and taxonomic synonyms. He is able to grab the list of the 100 most invasive species from the Global Invasive Species Database (http://www.issg.org/database/welcome/). He downloads both the EOL pages to extract the common names in French and the NCBI provider pages to get any taxonomic synonyms (first five species listed in Table 2 as example output).

**Table 2:** Output from the first five invasive species from Example 2

Species	French names	Taxonomic synonyms
*Ophiostoma ulmi*	None	None
*Eriocheir sinensis*	Crabe chinois à mitaines, Crabe chinois, Crabe velu, crabe poilu de Shangai	*Eriocheir chinensis*, *Eriocheir japonica sinensis*
*Vespula vulgaris*	None	*Paravespula vulgaris*
*Felis catus*	Chat	*Felis domesticus*, *Felis silvestris catus*
*Plasmodium relictum*	Paludisme des oiseaux	None

**Figure 3:**
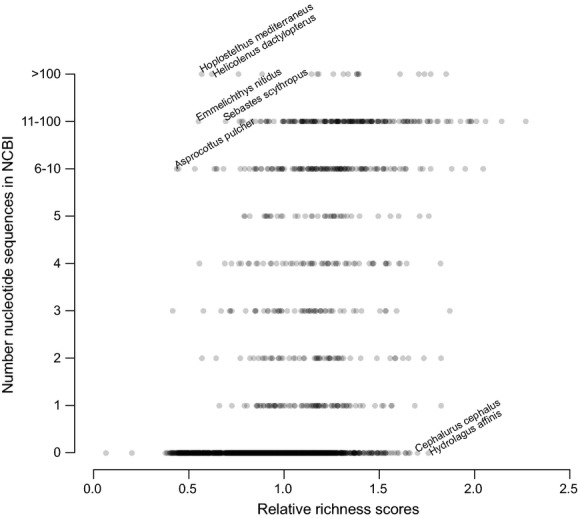
Comparing the relative EOL richness and the number of NCBI nucleotide sequences. Species in the lower right have a great deal of EOL information relative to the average deep water fish species (set to have a relative richness of 1.0) but have no nucleotide data at NCBI, while species in the upper left have many available sequences but underdeveloped EOL pages.

Example 3: A granting agency wants to know how much information is known about deep water (i.e., bathydemersal) fishes before they will fund exploratory research voyages. They download a list of fish species that inhabit deep water using *rfishbase* (Boettiger et al. 2013) and download the EOL pages using *Reol*. They gather the EOL richness score for each fish species and standardize it by the mean for all bathydemersal fish. They also gather the number of NCBI nucleotide sequences available for each of these species using *rentrez* (Winter 2013). They compare the relative EOL richness scores with the number of sequences as a proxy to find species that have much popular or curatorial interest (high richness scores) but have few or no sequences yet in NCBI (Fig. 3).

## Conclusions

EOL hosts over 3 million pages of information with data that comes in many forms (see http://eol.org/statistics). However, data would be difficult to extract by copy/pasting from the web, especially when there are a large number of taxa of interest; this approach has the advantage of automating repetitive tasks*. Reol* provides a tool for searching and amalgamating data on any number of species from a variety of sources. Writing simple batch scripts that can be rerun at any time will ensure up-to-date and fully reproducible results. Furthermore, *Reol* provides a novel tool for utilizing the taxonomic structuring that provider pages contribute to EOL, making it possible to take any set of taxa and create taxonomic trees based on their underlying relationships.

Examining patterns in data can be important. Users can determine whether their groups of interest are understudied (and potentially in which way), have a plethora of information, or see whether data are unequally distributed across taxa (both in terms of coverage and types of data). These differences might be attributed to community or curator interest (i.e., the page on pandas will draw more interest than a typical page on a marine fungus) but may also reflect patterns that are biologically important. For example, certain groups may have many name synonyms, suggesting a lack of coordination between taxonomists. It may also be possible to target areas where knowledge is lacking: There may be a group that is present in EOL but which has little information on its biogeography or extinction risk, but which based on trends in close relatives may be expected to be threatened.

*Reol* joins a growing body of R packages that interact with web-based APIs to streamline data acquisition. This is an important step forward in analyzing large publically available databases within a statistical framework.

## Supporting Information

**Appendix S1.** Code for Reol.
